# The Correlation Between Quality of Life and Positive Psychological Resources in Cancer Patients: A Meta-Analysis

**DOI:** 10.3389/fpsyg.2022.883157

**Published:** 2022-06-16

**Authors:** Xinxin Zhao, Siqi Tong, Ye Yang

**Affiliations:** ^1^Department of Hospice, ShengJing Hospital of China Medical University, Shenyang, China; ^2^Lymphedema Therapeutic Center, Liaoning Cancer Hospital and Institute, Cancer Hospital of China Medical University, Shenyang, China; ^3^Department of Ultrasound, ShengJing Hospital of China Medical University, Shenyang, China

**Keywords:** positive psychological resources, quality of life, cancer, meta-analysis, self-efficacy

## Abstract

**Purpose:**

This study aimed to assess the evidence of the association between positive psychological resources and quality of life in patients with cancer.

**Methods:**

Electronic searching was performed to retrieve articles from PubMed, Web of Science, Embase, CNKI, and CBM (from inception to 7 April 2022). Summary correlation coefficient (*r*) values were extracted from each study, and 95% CIs were calculated by the random-effect model. Subgroup and sensitivity analyses were performed to investigate potential heterogeneity.

**Results:**

In total, sixty-six articles were included in the present study. The pooled *r* for resilience was 0.71 (95%CI: 0.55, 0.87), hope 0.50 (95%CI: 0.43, 0.56), self-efficacy 0.53 (95%CI: 0.46, 0.61), self-esteem 0.46 (95%CI: 0.28, 0.63), and optimism 0.30 (95%CI: 0.19, 0.40). For subgroup analysis, no significant differences were found between minors and adults.

**Conclusion:**

This study indicated that resilience, hope, optimism, self-esteem, and self-efficacy were positively correlated with quality of life in patients with cancers. Therefore, intervention programs should be focused on increasing state-like positive psychological resources to improve the quality of life in patients with cancer.

## Introduction

Cancer is ranked as the major cause of death in countries with both more and less economic development, which is the most important barrier to enhancing life expectancy. Although medical anti-cancer therapies, radiation, and surgical oncology have made great progress, these, in turn, threaten patients’ mental health and quality of life. Previous research has found that patients with cancer have a more difficult time dealing with negative emotions and have a lower quality of life during the disease-free survivor stage (Chen et al., 2018; MacDonald et al., 2021; Perez-Tejada et al., 2021). This may be due to side effects of aggressive cancer therapy, financial hardship, difficulties in accessing affordable health insurance, and limited employment opportunities (Park, 2005; Kirchhoff et al., 2011; Nipp et al., 2017; Miller et al., 2019). Ultimately, patients with cancers are vulnerable to threats to their physical and psychological well-being. However, a longitudinal study (Lam et al., 2010) found that patients with cancer with high levels of psychological adaptation coped better with adversity during the disease, had fewer psychiatric disorders, and had a higher quality of life. Therefore, increasingly studies thought that positive psychological resources could help explain individual variation in the quality of life in patients with cancer.

Positive psychological resources have been associated with increasing attention to the oncology field in the past 30 years. Positive psychological resources include many constructs. Several state-like positive psychological constructs have been identified in the fields of positive psychology and psycho-oncology (Bao et al., 2017; Pitichat et al., 2018). The most common state-like constructs are resilience, hope, optimism, self-esteem, and self-efficacy in the context of cancer (Yang et al., 2014, 2016). These constructs are individuals’ positive psychological state of development and ability to maintain or restore relatively stable psychological and physical function when confronted with life-threatening events (Seiler and Jenewein, 2019). Therefore, this study focused on the role of resilience, hope, optimism, self-esteem, and self-efficacy in improving the quality of life among patients with cancer. Several studies found that patients with cancer with a high level of resilience were more able to cope with disease adjustment and maintain mental health (Park, 2005; Lam et al., 2010; Nipp et al., 2017). Resilience is considered as a developable capability characterized by a relatively stable psychological trait that reduces, adapts to, and even overcomes the destructive impacts caused by adverse factors in the face of disasters or stressors and a certain promoting role in alleviating the negative impact of traumatic pressure on individuals and maintaining the normal psychological state of the body (Richardson, 2002).

Snyder (Snyder et al., 1991; Snyder, 2000) conceptualized hope as a positive motivational state based on an inactively deprived sense of successful agency (achieving goals by available will power and determination) and pathways (pursuing goals by creating alternative routines). Optimism is a psychological trait that is considered to the degree of the general expectation that positive outcomes will happen rather than bad things (Carver et al., 2010; Carver and Scheier, 2014). Compared with pessimists, research on optimism stated that optimists were capable of adapting to and dealing with the negative impacts of cancer by accepting the reality, placing the light and humor among patients with cancer (Thieme et al., 2017). Furthermore, several studies have confirmed that general self-efficacy has a bearing on adjustment and management of patients with cancer (Fang et al., 2017; Hinz et al., 2019). In the cancer context, self-efficacy is defined as a positive belief in individual competence to deal with cancer and behaviors that occur during dealing with a cancer diagnosis, cancer treatment, and transitioning to patients with cancer (Luszczynska et al., 2005; Chirico et al., 2017). Self-esteem refers to an individual’s subjective evaluation of their worth and is derived from a person’s perceptions of self-evaluation about their self-competence and efficacy (Carpenito, 2017).

Quality of life is generally considered a multidimensional concept that includes physical, psychological, and social well-being, feelings of health and symptoms associated with illness or treatment (Felce and Perry, 1995). Quality of life is usually assessed subjectively by the patient, and if this is impossible, the assessment may be made by a doctor, nurse, or caregiver. It has already been an important indicator that monitors the process of cancer treatment and prognosis or rehabilitation effect in the recent years (Ferrell et al., 1998). At present, an extensive body of research has found that positive psychological resources are associated with quality of life and well-being of patients with cancer. For instance, Li et al. (2016) found that hope and resilience were positively associated with quality of life in adult patients with bladder cancer. Besides, Chung et al. (2021) suggested that greater resilience was associated with better quality of life and lower depressive symptoms, and Ho et al. (2021) found that self-esteem was significantly related to health-related quality of life in childhood patients with cancer. Other studies reported that patients with cancer with a high level of optimism and self-efficacy were likely to experience greater QoL (Wong and Fielding, 2007; Chu et al., 2021). However, a small number of studies thought that positive self-esteem and self-efficacy were not associated with quality of life (Mystakidou et al., 2013; Tonsing and Ow, 2018), or the association between them was very weak (Thieme et al., 2017; Vidthya et al., 2019). This variability in the included studies may be due to differences in demographic variables of participants, disease characteristics, measuring methods, and study quality.

In summary, the present study aimed to conduct a meta-analytic review that investigated associations among resilience, hope, optimism, self-esteem, self-efficacy, and quality of life in patients with cancer.

## Materials and Methods

### Study Selection and Procedures

According to the PRISM statement (Page et al., 2021), the present meta-analysis was performed and reported. We conducted an electronic search to retrieve articles from PubMed, Web of Science, Embase, CNKI, and CBM (from inception to 7 April 2022). In addition, relevant references were included by screening manually, and language was not limited. Our meta-analysis used these search terms: neoplasms, tumor, cancer, quality of life, resilience, hope, optimism, self-efficacy, and self-esteem. The PubMed search strategy is detailed in the Supplementary Material. This study included these articles that explored the association of positive psychological resources with quality of life in patients with cancer. However, case reports, review articles, comments, and letters were excluded from our study. Besides, this study excluded duplicated publications on the same study participants.

The primary data of eligible articles were extracted by three authors independently. The included primary data were the name of the first author, the year of publication, study location, sample size, the mean age of participants, cancer types, and the measuring instruments of quality of life and positive psychological resources, and Spearman and Pearson’s correlation coefficient (*r*).

The Joanna Briggs Institute (JBI) guidelines (The Joanna Briggs Institute Levels of Evidence and Grades of Recommendation Working Party: Supporting Document for the Joanna Briggs Institute levels of Evidence and Grades of Recommendation, 2014) were used to assess the study’s quality. The JBI guidelines contain 10 items: the purpose of the study, sampling method, characteristic description, reliability and validity of the tool, authenticity of the information, ethical issues, statistical analysis, statement of results, and research value. It is scored from 0 to 2 (0 = “not meeting the requirements,” 1 = “mentioned but not described in detail,” 2 = “detailed and comprehensive description”), and the total score ranges from 0 to 20. When the literature score is > the maximum score of 70%, it can be considered that the study quality is relatively high. Two authors evaluated the study quality of included articles, and the third author solved disagreements in this meta-analysis.

### Statistical Analysis

Heterogeneity was tested by the *Q* statistic (*P* < 0.05 = heterogeneity) and the inconsistency index (*I*^2^ > 50% = heterogeneity) (Higgins et al., 2003; Zamora et al., 2006; Leeflang et al., 2008), and publication was tested by the Egger method (Egger et al., 1997) (*p* < 0.05 = publication bias) and funnel plot (asymmetric plots = publication bias). A random-effect model was used rather than the fixed-effect model due to high heterogeneity (Wong and Fielding, 2007). Subgroup analysis was used to find whether effects were related to the factors as follows: participants’ group, the measuring instruments of quality of life, and positive psychological resources. In addition, sensitivity analysis was used to adjust for one possible atypical study.

R V4.0.2 was used to perform a meta-analysis in this study.

## Results

**FIGURE 1:**
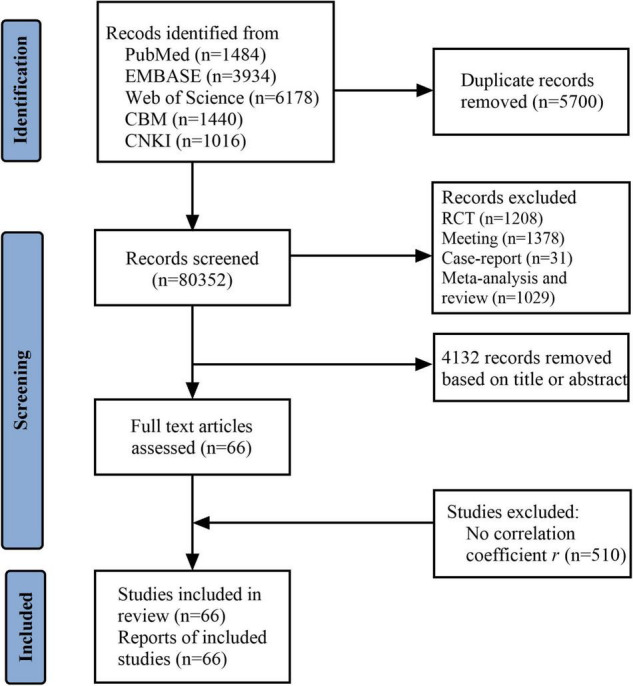
Study flow diagram.

Our meta-analysis retrieved 14,052 results. A total of 13,986 studies were excluded due to duplicate (5,700), review and meta-analysis (1,029), meeting (1,378), randomized controlled trial (1,208), and based on title and abstract (4,132) and full-text (510). Finally, there were 66 (Supplementary Material) articles that were included in this study (Figure 1). All the articles presented a clear research purpose, sufficient research basis, authentic information, correct statistical analysis method, appropriate and correct statement of analysis results, and research value. Only two articles adopted random sampling, and others used convenient sampling. We found that resilience, hope, optimism, and self-esteem were assessed by generic instruments in all included studies. As regards self-efficacy, ten out of 23 studies used specific instruments, including the Cancer Behavior Inventory (CBI), Cancer Survivors’ Self-Efficacy Scale (CSSES), Self-Efficacy Scale for Self-Management of Breast Cancer (SESSM-B), Symptom-Management Self-Efficacy Scale-Breast Cancer (SMSES-B), and Strategies Used by People to Promote Health (SUPPH). Besides, seven out of 66 studies involved minors. Cancer types mainly include breast, cervical, colorectal, lung, and prostate cancer. The sample size ranged from 39 to 953 (Table 1).

**TABLE 1:** Characteristics of included studies.

Study	Location	Sample size	Mean age (year)	Cancer type	Positive psychological resources (PPR)	Instruments for assessing PPR	Instruments for assessing QoL	Study quality
Johansson et al., 2018	Sweden	39	71.7	Colorectal	Self-efficacy	MFS	EORTC QLQ-C30	19
Koch et al., 2017	Germany	40	49.2	Breast	Self-esteem	RSES	FACT-B	19
Wu et al., 2015	Taiwan	40	16.4	Mixed	Resilience	RS	MMOL-AF	18
Colby and Shifren, 2013	United States	51	58.47	Breast	Optimism	LOT	FLIC	18
Haas, 2011	United States	73	60.12	Breast	Self-efficacy	PAAI	MQOL	19
Maeir et al., 2022	Israel	73	50.85	Mixed	Self-efficacy	NGSE	FACT-GP	18
Zheng and Pan, 2021	China	76	54.6	Cervical	Self-efficacy	SUPPH	FACT-G	18
Mystakidou et al., 2013	Greece	90	61.17	Breast	Self-efficacy	GSE	LASA	18
Finck et al., 2018	Germany	95	55.7	Breast	Optimism	LOT	EORTC QLQ-C30	19
Zhang et al., 2017	China	98	47.02	Breast	Resilience	CD-RISC	FACT-B	19
Clarke et al., 2019	United Kingdom	98	64	Head and neck	Resilience	CD-RISC	UW-QoL	18
Rammant et al., 2022	United States	99	63	Bladder	self-efficacy	GSE	FACT-BI	19
Chen et al., 2019	China	100	45.65	Cervical, endometrial, ovarian	Resilience	CD-RISC	FACT-G	18
Chu et al., 2021	United States	112	58.89	Breast	Self-efficacy	CBI	FACT-G	17
Kwak et al., 2017	South Korea	115	11–18	Mixed	Self-efficacy	SES	PedsQL 4.0	18
Zhao and Wang, 2019	China	118	67.45	Mixed	Resilience	RS-14	FACT-G	18
Wan, 2021	China	119	45.4	Breast	Resilience	CD-RISC	FACT-G	19
Liu et al., 2021	China	120	20–50	Breast	Resilience	CD-RISC	FACT-G	18
Shen et al., 2020	China	121	47.01	Breast	Hope, Self-efficacy	HHI, GSES	FACT-B	17
Zhong et al., 2019	China	124	49.54	Breast	Resilience	CD-RISC	FACT-B	19
Sjoquist et al., 2013	Australia	126	62.1	Ovarian	Hope	HHI	FACT-G	18
Li et al., 2019	China	128	46.89	Breast	Resilience	CD-RISC	FACT-B	18
Tong, 2020	China	128	64.7	Gastric	Self-efficacy	GSES	SQLI	19
Young et al., 2014	South Korea	129	52.09	Breast	Resilience	RS	FACT-B	18
Liu, 2021	China	129	56.4	Colorectal	Resilience	CD-RISC	FACT-G	18
Tonsing and Ow, 2018	Singapore	129	17.3	Mixed	Self-esteem	RSES	QoL-CSS	17
Perez-Tejada et al., 2021	Spain	134	54.4	Breast	Self-esteem	RSES	QLACS	19
Xu et al., 2021	China	134	52.6	Breast	Resilience	CD-RISC	FACT-B	18
Choi et al., 2022	South Korea	136	58.8	Gastric	Self-esteem	SES	QOL-CS	18
Chung et al., 2021	Hong Kong	138	10.6	Leukemia or Brain tumor or Lymphoma	Resilience, self-esteem	RS-10, RSES	PedsOL 3.0	18
Park et al., 2021	South Korea	140	48.9	Breast	Self-efficacy	SESSM-B	FACT-B	17
Luo et al., 2022	China	146	6.2	Mixed	Resilience	CD-RISC	SF-6D	19
Zhang et al., 2015	China	147	57.51	Lung	Self-efficacy	SUPPH	FACT-L	18
Lee et al., 2001	Taiwan	150	44.8	Breast	Self-esteem	RSES	OLI-cancer	17
Jiao et al., 2020	China	160	18 +	Colorectal	Resilience	CD-RISC	EORTC QLQ-C30	18
Mazanec et al., 2010	Frances	163	58.24	Mixed	Optimism	LOT	FACT-G	17
Zhao D. J. et al., 2020	China	172	57.3	Lung	Resilience	CD-RISC	FACT-L	18
Ho et al., 2021	Hong Kong	176	12.56	Mixed	Hope, self-esteem	HHI, RSES	PedsOL 4.0	19
Hu et al., 2016	China	193	67.38	Liver cancer	Hope	HHI	FACT-Hep	17
Ye et al., 2014	China	193	55.46	Mixed	Hope	HHI	FACT-G	18
Sharif Nia et al., 2021	Iran	200	51.31	Mixed	Hope	AHS	EORTC QLQ-C30	18
Chin et al., 2021	Taiwan	201	53.6	Breast	Self-efficacy	SMSES-B	EORTC QLQ-C30	19
Groarke et al., 2020	United Kingdom	204	65.24	Prostate	Resilience	CD-RISC	PORPUS-P	18
Bo et al., 2019	South Korea	204	54.2	Mixed	Self-efficacy	CSSES	SF-36	18
Wang et al., 2017	China	206	54.6	Mixed	Self-efficacy	GSES	QOL-CS	17
Martins et al., 2018	Portugal	211	13.29	Mixed	Hope	CHS	DCGM-12	19
Yu et al., 2021	China	217	56.24	Breast	Self-efficacy	SUPPH	FACT-B	18
Wu et al., 2013	China	224	47.54	Breast	Hope	HHI	FACT-B	17
Zhang et al., 2020	China	230	56.13	Oral	Hope	HHI	FACT-H&N	19
Zhou et al., 2022	China	231	48.15	Breast	Resilience	CD-RISC	FACT-B	18
Zhao Y. Q. et al., 2020	China	237	18 +	Cervical	Self-efficacy	SUPPH	FACT-Cx	18
Yeung and Lu, 2014	China	238	55.7	Mixed	Self-efficacy	CBI	QOL-CS	17
Gong et al., 2016	China	265	50.24	Cervical	Hope	HHI	WHOQOL-BREF	18
Chen et al., 2017	China	273	46.91	Breast	Resilience	CD-RISC	QOL-CS	17
He et al., 2019	China	284	49	Lung, breast and liver	Resilience	CD-RISC	EORTC QLQ-C30	18
McAteer and Gillanders, 2019	United Kingdom	286	67	Prostate	Self-esteem	MSES	FACT-G	19
Sharour et al., 2019	Jordan	320	51.9	Colorectal	Hope	HHI	FACT-C	19
Wong and Fielding, 2007	Hong Kong	334	64.66	Lung	Optimism	LOT	FACT-G	18
Omran and Mcmillan, 2018	United States	341	57.9	Mixed	Self-efficacy	CBI	MQOL-C	18
Thieme et al., 2017	Germany	354	61.2	Breast and gynecological	Optimism, self-efficacy	LOT, GSES	EORTC QLQ-C30	17
Li et al., 2016	China	365	63.76	Bladder	Resilience, hope	RS-14, AHS	FACT-BL	19
Yuan et al., 2022	China	404	67.06	Prostate	self-efficacy	GSES	FACT-P	18
Schofield et al., 2016	Australia	429	67	Metastatic colorectal	Hope	SHS, LOT	EQ-5D	18
Li et al., 2021	China	450	47.76	Breast	Hope	HHI	FACT-B	17
Chen et al., 2018	China	452	62.1	Lung	Self-efficacy	GSES	SF-36	19
Vidthya et al., 2019	Malaysia	953	46.45	Mixed	Self-esteem	RSES	WHOQOL-BREF	17

*AHS, Adult Hope Scale; CBI, Cancer Behavior Inventory; CD-RISC, Connor-Davidson resilience scale; CHS, Children’s Hope Scale; CSSES, Cancer Survivors’ Self-Efficacy Scale; GSE, General Self-efficacy beliefs Scale; HHI, Herth Hope Index; LOT, Life Orientation Test; MFS, Maintain Function Scale; MSES, Masculine Self-Esteem Scale; NGSE, New General Self-Efficacy Scale; PAAI, Physical Activity Assessment Inventory; RSES, Rosenberg Self-Esteem Scale; SES, Self-Efficacy-Scale; SESSM-B, Self-Efficacy Scale for Self-Management of Breast Cancer; SHS, State Hope Scale; SMSES-B, Symptom-Management Self-Efficacy Scale-Breast Cancer; SQLI, Spitzer Quality of Life Index; SUPPH, Strategies Used by People to Promote Health.*

*GCGM, DISABKIDS Chronic Generic Measure; EORTC QLQ, European Organization for Research and Treatment of Cancer quality of life, Questionnaire; EQ-5D, European Quality of Life-5 Dimensions; FACT-B, Functional Assessment of Cancer Therapy Breast cancer; FACT-BL, Functional Assessment of Cancer Therapy-Bladder; FACT-C, Functional Assessment of Cancer Therapy-Colorectal cancer; FACT-G, Functional Assessment of Cancer Therapy-General; FACT-H&N, Functional Assessment of Cancer Therapy-Head and Neck; FACT-Hep, Functional Assessment of Cancer Therapy-Hepatobiliary; FACT-L, Functional Assessment of Cancer Therapy-Lung; FLIC, Functional Living Index-Cancer; LASA, Linear Analog Scale Assessment; MMQL-AF, Minneapolis-Manchester Quality of Life Instrument-Adolescent Form; MQOL, McGill Quality of Life Questionnaire; MQOL-C, Multidimensional Quality of Life Scale-Cancer; PedsQL, Pediatric Quality-of-Life; QLACS, Quality of Life in Adult Cancer Survivors; QLI, Quality-of-Life Index; QOL-CS, Quality of Life-Cancer Survivors; SF, Short Form; UW-QoL, University of Washington quality of life; WHOQOL-BREF, World Health Organization’ s Quality of Life Questionnaire-Brief.*

### Resilience and Quality of Life

**FIGURE 2:**
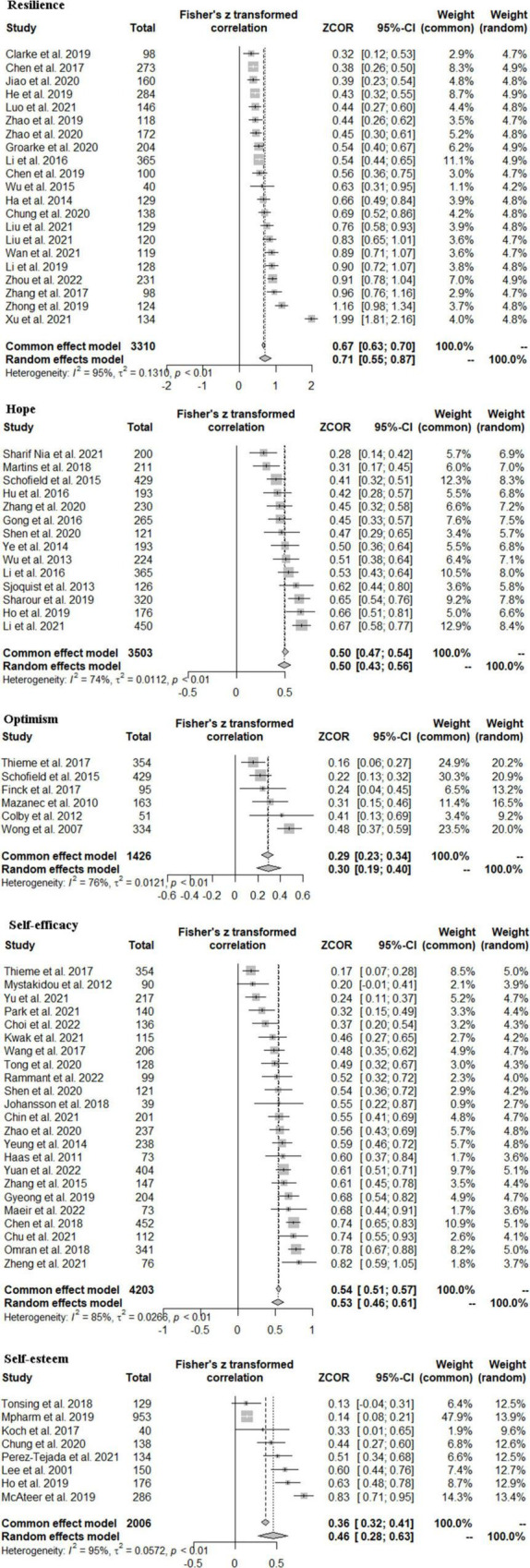
Effect sizes of the correlation between five positive psychological resources and quality of life.

In total, twenty-one studies involving 3,310 patients with cancer examined the association of resilience with quality of life in patients with cancer. The meta-analysis of these studies found a large and significant overall effect size of *r* = 0.71 with CIs excluding zero (95%CI: 0.55, 0.87) (Figure 2). In addition, the results of the subgroup analysis indicated that the impact of resilience on quality of life was not different between minors (*k* = 3, *r* = 0.58, 95%CI: 0.40, 0.76) and adults (*k* = 18, *r* = 0.73, 95%CI: 0.54, 0.91) (Table 2 and Supplementary Figure A).

**TABLE 2:** Correlations between positive psychological resources and quality of life in cancer patients: subgroup analysis.

Subgroup	*k*	*r* (95%CI)	*I*^2^ (%)	*P*-value
**Resilience**				
Participants				0.251
Minors	3	0.58 (0.40, 0.76)	58.0	
Adults	18	0.73 (0.54, 0.91)	95.5	
**Hope**				
Participants				0.934
Minors	2	0.48 (0.14, 0.83)	91.5	
Adults	12	0.50 (0.43, 0.56)	70.9	
Specific instruments for quality of life				0.123
No	2	0.43 (0.35, 0.50)	0.0	
Yes	12	0.51 (0.43, 0.58)	75.2	
**Self-efficacy**				
Specific instruments for self-efficacy				0.228
No	13	0.49 (0.39, 0.59)	85.5	
Yes	10	0.58 (0.47, 0.70)	84.1	
Specific instruments for quality of life				0.053
No	4	0.52 (0.43, 0.60)	53.4	
Yes	19	0.65 (0.54, 0.76)	84.9	
**Self-esteem**				
Participants				0.659
Minors	3	0.40 (0.12, 0.69)	89.1	
Adults	5	0.49 (0.24, 0.73)	96.6	

### Hope and Quality of Life

The meta-analysis of fourteen studies involving 3,503 patients with cancer, revealed a large and significant overall effect size of *r* = 0.50 with CIs excluding zero (95%CI: 0.43, 0.56) (Figure 2). In subgroup analysis, no differences were found between minors’ group (*k* = 2, *r* = 0.48, 95%CI: 0.14, 0.83) and adults’ group (*k* = 12, *r* = 0.50, 95%CI: 0.43, 0.56) (Table 2 and Supplementary Figure A). In addition, the results of subgroup analysis indicated that the impact of resilience based on a quality-of-life specific scale (*k* = 12, *r* = 0.51, 95%CI: 0.43, 0.58) was similar to studies based on a generic scale (*k* = 2, *r* = 0.43, 95%CI: 0.35, 0.50) (Table 2 and Supplementary Figure B).

### Optimism and Quality of Life

In total, six studies, involving 1,426 patients with cancer, examined the relationship between self-esteem and quality of life, and yielded a significant and medium overall effect size of *r* = 0.30 with all CIs excluding zero (95%CI: 0.19, 0.40) (Figure 2) and thus statistically significant.

### Self-Efficacy and Quality of Life

In total, twenty-three studies involving 4,203 patients with cancer examined the association between self-efficacy and quality of life. The current meta-analysis of these studies yielded a significant and large effect size of *r* = 0.53 with CIs excluding zero (95%CI: 0.46, 0.61) (Figure 2). Besides, there were no differences in instruments for assessing self-efficacy (*p* = 0.228) and quality of life (*p* = 0.053) (Table 2 and Supplementary Figures B,C).

### Self-Esteem and Quality of Life

In total, eight studies, involving 2,006 patients with cancer, explored the relationship between self-esteem and quality of life and yielded a significant and medium overall effect size of *r* = 0.46 with confidence intervals excluding zero (95%CI: 0.28, 0.63) (Figure 2). Differences were not found between minor participants and adult participants (*p* = 0.659) (Table 2 and Supplementary Figure A).

### Sensitivity Analysis and Publication Bias

**FIGURE 3:**
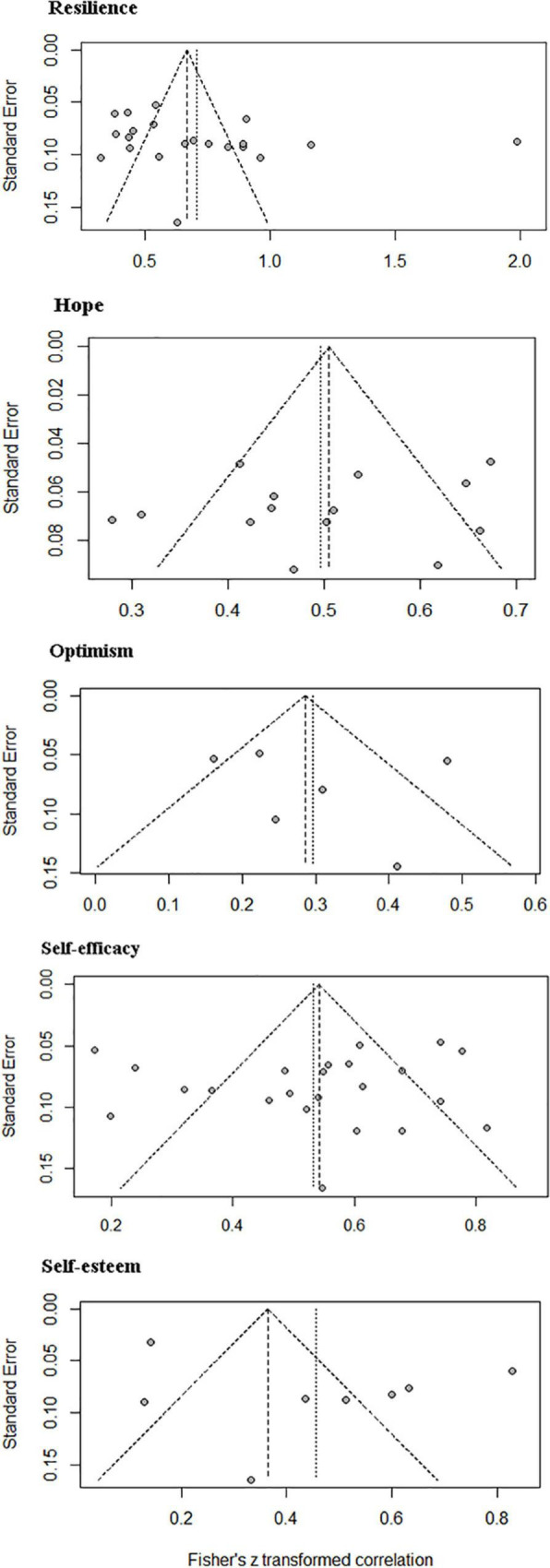
Publication bias based on the funnel plots.

Sensitivity analysis showed that all the pooled *r* for quality of life in patients with cancer were stable, which indicated that our results were reliable. The result of the Egger test indicated that there was no publication bias in this meta-analysis (resilience: *p* = 0.226; hope: *p* = 0.420; self-efficacy: *p* = 0.660). The Egger test of optimism and self-esteem was not analyzed due to the small number of studies (*n* < 10). Figure 3 presents the funnel plot.

## Discussion

The present study aimed to conduct a meta-analysis of the empirical literature on the association of state-like positive psychological resources with quality of life among patients with cancer. The current study focused on five state-like positive psychological variables (resilience, hope, optimism, self-efficacy, and self-esteem) and quality of life of patients with cancer. The meta-analysis showed that quality of life of patients with cancer was positively and significantly associated with resilience, hope, optimism, self-efficacy, and self-esteem, which were consistent with previous studies (Wong and Fielding, 2007; Li et al., 2016; Chu et al., 2021; Chung et al., 2021; Ho et al., 2021).

Patients with cancer face multiple adversities within their illness, including both the physical impact of the disease, such as pain and discomfort, and treatment, as well as the psychological aspects of the knowledge of having a potentially life-threatening illness, which thereby causes poor quality of life during cancer treatment. Generally, resilience, hope, optimism, self-efficacy, and self-esteem are both stable, state-like positive psychological variables that can act as protective factors against adversity to improve quality of life. Protective factors are circumstances or attributes that help individuals deal more effectively with stressful events. These five state-like positive psychological constructs can act as protective factors against the subjective experience of hardship, specifically of receiving a potentially adverse cancer diagnosis. These variables may help patients with cancer overcome and/or become proactive in the context of cancer diagnosis and treatment by making patients more confident in coping with cancer, and less susceptible to negative mood states that negatively impact quality of life. These findings were supported by prior evidence (Coopersmith, 1967; Snyder et al., 1991; Martínez-Correa et al., 2006; Duggal et al., 2016).

Specifically, evidence suggests that high-resilient people strategically elicit positive emotions through the use of humor, relaxation techniques, and optimistic thinking to proactively cultivate their positive emotions (Tugade and Fredrickson, 2004). Then, positive emotionality emerges as the crucial element of resilience. A study found that resilience had an indirect impact on quality of life of patients with cancer by influencing social support (Zhang et al., 2017). According to this theory of Snyder, hope is conceptualized as a positive motivational state based on an interactively derived sense of successful agency (goal-directed energy) and pathways (planning to meet goals) (Snyder et al., 1991; Snyder, 2000). Rousseau (2000) found that hope could be developed by learning to control one’s symptoms, exploring one’s faith, and strengthening interpersonal relationships. Another study found that highly hopeful individuals reported significantly higher levels of personal adjustment and global life satisfaction, and less psychological distress (Gilman et al., 2006). An optimistic attitude plays a crucial role in effectively coping with disease diagnosis, treatment, prognosis, and in enhancing the quality of life (Rousseau, 2000). Furthermore, compared with pessimists, optimistic patients with cancer reported greater survival rates a year after diagnosis (Allison et al., 2003). Highly efficacious patients with cancer characterized by a sense of agency or control may perceive some causal relationship between coping behaviors executed and certain desired outcomes, including the level of the type of quality of life (Merluzzi et al., 2001). Andrea et al. conducted a meta-analysis involving 3,162 patients with cancer and found that there was a large significant overall effect size of *r* = 0.73 for the association of self-efficacy with quality of life (Chirico et al., 2017). Low self-esteem patients mean self-contempt, self-disappointment, self-rejection, and lack of self-respect for themselves. Based on the theory of Rosenberg (1965), a high level of self-esteem implies that they have high self-respect for who they are within limits, and do not assume that they are superior in any way to anyone else. A cancer diagnosis tends to affect the patients’ body image, which negatively causes changes in self-esteem. Even the easiest daily routine may be disrupted, and patients need to be aware of these long-term consequences that negatively influence their quality of life and mental health.

In this study, subgroup analysis indicated that no differences were found in participant groups (minors and adults), and measuring instruments (generic and specific instruments). However, these state-like variables and integration processes in patients with cancer are significantly different from those in other populations due to the complex treatment and potential fatality in the former. Moreover, the current evaluation scales are based on participants with depression and anxiety or the general population (Carver et al., 2010; Carver and Scheier, 2014). For instance, the Connor Davidson Resilience Scale (CD-RISC) was developed based on a sample with anxiety symptoms. In this study, only self-efficacy was used with specific instruments to assess the level of self-efficacy in patients with cancer. Therefore, it is crucial to develop scales specifically for patients with cancer to gauge their mental health with greater reliability. Besides, most studies investigated the effect of optimism on quality of life in adult patients with cancer, but few studies examined the association between children and adolescent patients. Hence, future studies can benefit from examining the impact of optimism on the quality of life in minors with cancer.

### Limitations

The present meta-analysis has some limitations. At first, there is a dearth of longitudinal studies on positive psychological resources and quality of life among patients with cancer. Although a cross-sectional study is easier to be performed, a longitudinal study is more valuable in terms of answering questions concerning the long-term relations between positive psychological resources and quality of life in patients with cancer. Second, the subgroup analysis of the children or adolescent group and the adult group could not be performed for every meta-analysis in this study due to the limited number of studies concerning the association of self-efficacy and optimism with quality of life among patients with cancer. Therefore, there is a need for additional research on the impact of self-efficacy and optimism on quality of life in children or adolescents with cancer. Besides, although this study has investigated each positive psychological resource individually, it is significant to recognize that some positive psychological variables may co-vary. For instance, highly hopeful individuals tend to effectively buffer the impact of stressful and negative life events, and successfully reach their end goals, so they possess more resilience (Ong et al., 2006). Thus, hope might trump the effects of resilience on quality of life. Besides, the optimistic attitude inherent in hopeful individuals plays a crucial role in improving health-related quality of life. Therefore, further research needs to examine their covariation and unique and interactive relation with quality of life among patients with cancer. Finally, most studies were written in English in this study, which may lead to language bias. However, Thornton and Lee thought that there was a similar bias in all meta-analyses that did not review all studies. Therefore, despite, this limitation, this study’s outcomes are reliable and warranted.

### Clinical Implications

Although these five stake-like variables are defined in different models, they may affect mental health and quality of life among patients with cancer by different mechanisms, these variables have two significant points in common. Namely, these constructs are positive coping with styles or protective factors to fight cancers and they are dynamic and developmental resources. Therefore, these variables could be increased through intervention to better improve the quality of life in patients with cancer. Furthermore, increasing research has found that interventions based on positive psychological resources could cope with mental problems and enhance the quality of life in patients with cancer. For example, in a randomized and controlled trial, Promoting Resilience in Stress Management (PRISM), a psychosocial intervention for adolescents and young adults with cancer, enhances resilience resources *via* four skills-based training sessions, compared with the usual care (UC) may improve health-related quality of life, especially in psychosocial domains of well-being (Steineck et al., 2019). Berg et al. (2020) developed and tested Achieving Wellness After Kancer in Early life (AWAKE), a scalable 8-week app-based program consisting of educational videos, mood/activity tracking, and telephone-based coaching to promote hope and quality of life in young adult cancer survivors, which evidence that the AWAKE supports patients cope with cancer-related sequelae and reestablish goals across life domains after experiencing cancer. Evidence suggests that nurse-administered self-efficacy interventions given on five monthly occasions and designed to enhance patients’ self-care self-efficacy have significantly higher scores on quality of life and self-care self-efficacy than the control group and significantly less symptom distress (Lev et al., 2001). Besides, beauty care interventions (Richard et al., 2019) and Framed Portrait Experience interventions (Saita and Acquati, 2020) are similar to enhancing self-esteem and self-efficacy among patients with cancer. Therefore, interventions based on state-like positive psychological constructs should be emphasized and developed in the field of oncology psychology to enhance the quality of life.

## Conclusion

The current meta-analysis provided a comprehensive summary of the current literature on state-like positive psychological constructs and quality of life in patients with cancer. Results of this study indicated that state-like variables, including resilience, hope, optimism, self-esteem, and self-efficacy were positively correlated with quality of life in patients with cancer. Therefore, intervention programs should be focused on increasing state-like positive psychological resources to improve the quality of life in patients with cancer.

## Data Availability Statement

The original contributions presented in this study are included in the article/Supplementary Material, further inquiries can be directed to the corresponding author.

## Author Contributions

XZ searched databases, selected studies, extracted information, assessed study quality, analyzed data, drafted, and revised manuscript. ST contributed to study selection, data extraction, and quality assessment. XZ and YY assessed study and revised manuscript. YY conceived the hypothesis, developed the study methods, and revised the manuscript. All authors contributed to the article and approved the submitted version.

## Conflict of Interest

The authors declare that the research was conducted in the absence of any commercial or financial relationships that could be construed as a potential conflict of interest.

## Publisher’s Note

All claims expressed in this article are solely those of the authors and do not necessarily represent those of their affiliated organizations, or those of the publisher, the editors and the reviewers. Any product that may be evaluated in this article, or claim that may be made by its manufacturer, is not guaranteed or endorsed by the publisher.
