# Supplementary material for: The Correlation Between Quality of Life and Positive Psychological Resources in Cancer Patients: A Meta-Analysis
Source: Front Psychol. 2022 Jun 16;13:883157. doi: 10.3389/fpsyg.2022.883157 (PMC9245894; doi:10.3389/fpsyg.2022.883157)

**Search strategy-PubMed**

((("Neoplasms"[Mesh]) OR (((((((((((((((((((((((Tumor[Title/Abstract]) OR (Neoplasm[Title/Abstract])) OR (Tumors[Title/Abstract])) OR (Neoplasia[Title/Abstract])) OR (Neoplasias[Title/Abstract])) OR (Cancer[Title/Abstract])) OR (Cancers[Title/Abstract])) OR (Malignant Neoplasm[Title/Abstract])) OR (Malignancy[Title/Abstract])) OR (Malignancies[Title/Abstract])) OR (Malignant Neoplasms[Title/Abstract])) OR (Neoplasm, Malignant[Title/Abstract])) OR (Neoplasms, Malignant[Title/Abstract])) OR (Benign Neoplasms[Title/Abstract])) OR (Benign Neoplasm[Title/Abstract])) OR (Neoplasms, Benign[Title/Abstract])) OR (Neoplasm, Benign[Title/Abstract])) OR (oncology[Title/Abstract])) OR (leukemia[Title/Abstract])) OR (lymphoma[Title/Abstract])) OR (myeloma[Title/Abstract])) OR (myelodysplastic syndrome[Title/Abstract])) OR (MDS[Title/Abstract]))) AND (("Quality of Life"[Mesh]) OR ((((Life Quality[Title/Abstract]) OR (Health-Related Quality Of Life[Title/Abstract])) OR (Health Related Quality Of Life[Title/Abstract])) OR (HRQOL[Title/Abstract])))) AND (((((("Optimism"[Mesh]) OR (((Positive Attitude[Title/Abstract]) OR (Attitude, Positive[Title/Abstract])) OR (Positive Thinking[Title/Abstract]))) OR (("Self Efficacy"[Mesh]) OR ((Efficacy, Self[Title/Abstract]) OR (self-efficacy[Title/Abstract])))) OR (("Hope"[Mesh]) OR ((Hopes[Title/Abstract]) OR (Hopefulness[Title/Abstract])))) OR (("Resilience, Psychological"[Mesh]) OR ((((Psychological Resilience[Title/Abstract]) OR (Resiliency, Psychological[Title/Abstract])) OR (Psychological Resiliency[Title/Abstract])) OR (resilience[Title/Abstract])))) OR (((((Self Esteem[Title/Abstract]) OR (Esteem, Self[Title/Abstract])) OR (Self Esteems[Title/Abstract])) OR (Self-esteem[Title/Abstract])) OR (Esteem[Title/Abstract])))

**Following are 66 included articles in our study.**

1. Johansson AC, Brink E, Cliffordson C, Axelsson M. The function of fatigue and illness perceptions as mediators between self-efficacy and health-related quality of life during the first year after surgery in persons treated for colorectal cancer. J Clin Nurs. 2018, 27(7-8): e1537-e1548.
2. Koch AK, Rabsilber S, Lauche R, Kümmel S, Dobos G, Langhorst J, Cramer H. The effects of yoga and self-esteem on menopausal symptoms and quality of life in breast cancer survivors-A secondary analysis of a randomized controlled trial. Maturitas. 2017, 105:95-99.
3. Wu WW, Tsai SY, Liang SY, Liu CY, Jou ST, Berry DL. The mediating role of resilience on quality of life and cancer symptom distress in adolescent patients with cancer. J Pediatr Oncol Nurs. 2015, 32(5):304-13.
4. Colby DA, Shifren K. Optimism, mental health, and quality of life: a study among breast cancer patients. Psychol Health Med. 2013, 18(1):10-20.
5. Haas BK. Fatigue, self-efficacy, physical activity, and quality of life in women with breast cancer. Cancer Nurs. 2011, 34(4):322-34.
6. Maeir T, Nahum M, Makranz C, Tsabari S, Peretz T, Gilboa Y. Predictors of quality of life among adults with self-reported cancer related cognitive impairment. Disabil Rehabil. 2022, 17:1-7.
7. Zheng HX, Pan C. Effects of negative emotional self-efficacy and social support on quality of life of postoperative patients with cervical cancer. Maternal and Child Health Care of China. 2021, 36(14):3335-3339.
8. Mystakidou K, Tsilika E, Parpa E, Gogou P, Panagiotou I, Vassiliou I, Gouliamos A. Relationship of general self-efficacy with anxiety, symptom severity and quality of life in cancer patients before and after radiotherapy treatment. Psychooncology. 2013, 22(5):1089-95.
9. Finck C, Barradas S, Zenger M, Hinz A. Quality of life in breast cancer patients: Associations with optimism and social support. Int J Clin Health Psychol. 2018,18(1):27-34.
10. Clarke G, Asiedu YA, Herd K, Sharma S. Exploring the relation between patients' resilience and quality of life after treatment for cancer of the head and neck. Br J Oral Maxillofac Surg. 2019;57(10):1044-1048.
11. Zhang H, Zhao Q, Cao P, Ren G. Resilience and Quality of Life: Exploring the Mediator Role of Social Support in Patients with Breast Cancer. Med Sci Monit. 2017, 23:5969-5979.
12. Rammant E, Leung TM, Gore JL, Berry D, Given B, Lee CT, Quale D, Mohamed NE. Associations of self-efficacy, social support and coping strategies with health-related quality of life after radical cystectomy for bladder cancer: A cross-sectional study. Eur J Cancer Care (Engl). 2022, 18. 10.1111/ecc.13571.
13. Chen HP, Gao LL, Jiang MT, Zheng X. The correlation research on resilience and the quality of life among postoperative patients with gynecological malignant oncology in chemotherapy. Journal of Nursing Administration. 2019,19(11):799-802.
14. Chu Q, Wong CCY, Chen LJ, Shin LJ, Chen LQ, Lu Q. Self-stigma and quality of life among Chinese American breast cancer survivors: A serial multiple mediation model. Psycho-Oncology. 2021, 30(3):392-399.
15. Kwak Y, Kim Y, Choi ES, Im HJ. Self-efficacy, post-traumatic growth, and quality of life of pediatric cancer survivors: A cross-sectional study. European journal of oncology nursing: the official journal of European Oncology Nursing Society. 2021, 54:102019.
16. Zhao XX, Wang YM. Study on the relationship between quality of life and social support and psychological resilience in elderly patients with end-stage cancer. Modern Oncology. 2019, 27(05):852-855.
17. Wan M. Correlation and Influencing Factors of Psychological Resilience and Quality of Life in Patients with Breast Cancer during Chemotherapy. Medical Inovation of China. 2021, 18(28):142-145.
18. Liu F, Pang JM, Wang XY, Wang Y. The mediating effect of psychological resilience of breast cancer patients on reproductive concern and quality of life. Modern Clinical Nursing. 2021, 20(12):7-12.
19. Shen A, Qiang W, Wang Y, Chen Y. Quality of life among breast cancer survivors with triple negative breast cancer--role of hope, self-efficacy and social support. Eur J Oncol Nurs. 2020, 46:101771.
20. Zhong MS, Li XB, Tang N, Chen X, Fan TT. Role of resilience in relationship between perceived social support and quality of life of breast cancer patients. Chinese Nursing Research. 2019, 33(02):237-240.
21. Sjoquist KM, Friedlander ML, O'Connell RL, Voysey M, King MT, Stockler MR, Oza AM, Gillies K, Martyn JK, Butow PN. Hope, quality of life, and benefit from treatment in women having chemotherapy for platinum-resistant/refractory recurrent ovarian cancer: the gynecologic cancer intergroup symptom benefit study. Oncologist. 2013, 18(11):1221-8.
22. Li TX, Tan HH, Chen Y, Jiang JF, Xiong M. Analysis the relationship between psychological resilience and quality of life and its influencing factors in breast cancer patients during postoperative chemotherapy. Oncol Prog. 2019, 17(19):2343-2347.
23. Tong XJ. Mediating and regulating effects of self-efficacy on health literacy and quality of life in patients with gastric cancer undergoing chemotherapy. WCJD. 2020, 28(05):167-171.
24. Liu GL. Correlation between psychological resilience and quality of life in patients with rectal cancer after radiotherapy. Medical higher vocational education and modern nursing. 2021, 4(06):522-526.
25. Tonsing KN, Ow R. Quality of Life, Self-Esteem, and Future Expectations of Adolescent and Young Adult Cancer Survivors. Health Soc Work. 2018, 43(1):15-21.
26. Young HB, Jung JE, Young CS. Effects of Resilience, Post-traumatic stress disorder on the Quality of Life in Patients with Breast Cancer. Korean Journal of Women Health Nursing. 2014, 20(1):83.
27. Perez-Tejada J, Aizpurua-Perez I, Labaka A, Vegas O, Ugartemendia G, Arregi A. Distress, proinflammatory cytokines and self-esteem as predictors of quality of life in breast cancer survivors. Physiol Behav. 2020, 230:113297.
28. Xu Y, Zhang HY, Jiang CY, Yan YW. Correlation among supportive care needs, psychological resilience and quality of life in breast cancer patients receiving postoperative chemotherapy. Chin J Breast Dis. 2021,15(06):352-358.
29. Choi J, Kim S, Choi M, Hyung WJ. Factors affecting the quality of life of gastric cancer survivors. Supportive Care in Cancer 2022, 30(4):3215-3224.
30. Chung JOK, Li WHC, Cheung AT, Ho LLK, Xia W, Chan GCF, Lopez V. Relationships among resilience, depressive symptoms, self-esteem, and quality of life in children with cancer. Psycho-oncology. 2020 Sep 11.
31. Park JH, Jung YS, Kim JY, Bae SH. Determinants of quality of life in women immediately following the completion of primary treatment of breast cancer: A cross-sectional study. PLoS ONE. 2021, 16(10 October).
32. Luo YH, Li WHC, Cheung AT, Ho LLK, Xia W, He XL, Zhang JP, Chung JOK. Relationships between resilience and quality of life in parents of children with cancer. J Health Psychol. 2022, 27(5):1048-1056.
33. Zhang SM, Gu W, Gao M, Chen P. Self-efficacy as a mediator between social support and quality of life in lung cancer patients receiving chemotherapy. China Journal of Health Psychology. 2015, 23(9).
34. Lee HL, Ku NP, Dow WJ, Pai L. Factors related to quality of life in breast cancer patients receiving chemotherapy. J Nurs Res. 2001, 9(3):57-68.
35. Jiao TT, Li L, Wang ZX. Correlation between resilience and quality of life in patients with colon cancer. Today nurse. 2020, 27(03):15-17.
36. Mazanec SR, Daly BJ, Douglas SL, Lipson AR. The relationship between optimism and quality of life in newly diagnosed cancer patients. Cancer Nurs. 2010, 33(3):235-43.
37. Zhao DJ, Kang SR, Hao N, Wu R, Mo LL, Xiao YH. Evaluation of Ｒesilience and Quality of Life in Patients with Advanced Lung Cancer and Their relationship. J Cancer Control Treat. 2020, 33(08):679-684.
38. Ho L, Li W, Cheung AT, Ho E, Lam K, Chiu SY, Chan G, Chung J. Relationships among hope, psychological well-being and health-related quality of life in childhood cancer survivors. J Health Psychol. 2019, 17:1359105319882742.
39. Hu SH, Wang WL, Zuo XF, Zhang ZH, Zhang CJ, Ren CX. Mediating effect of aspiration level on the relationship between family function and quality of life among elderly patients with hepatocellular carcinoma. Chinese Journal of Clinical Psychology. 2016, 24(3).
40. Ye ZY, Gao XX, Cheng YQ. The relationship between hope, coping style and quality of life among advanced cancer patients. Chinese Nursing Management. 2014, 14(1).
41. Sharif Nia H, Lehto RH, Seyedfatemi N, Mohammadinezhad M. A path analysis model of spiritual well-being and quality of life in Iranian cancer patients: a mediating role of hope. Supportive Care in Cancer 2021, 29(10):6013-6019.
42. Chin CH, Tseng LM, Chao TC, Wang TJ, Wu SF, Liang SY. Self-care as a mediator between symptom-management self-efficacy and quality of life in women with breast cancer. PLoS One. 2021, 16(2):e0246430.
43. Groarke A, Curtis R, Skelton J, Groarke JM. Quality of life and adjustment in men with prostate cancer: Interplay of stress, threat and resilience. PLoS One. 2020, 15(9): e0239469.
44. Bo GL, Lee TS, Kim SH. Mediation Effect of Self-Efficacy on the Relationship between Perceived Self-Management Support and Health-Related Quality of Life among Cancer Survivors. Journal of Korean Academy of Nursing. 2019, 49(3):298.
45. Wang Q, Zhang XQ, Wang QP. The specialties of illness perception, and its connection with self-efficacy and quality of life in cancer chemotherapy patients. Anhui Medical and Pharmaceutical Journal. 2017, 21(8).
46. Martins AR, Crespo C, Salvador Á, Santos S, Carona C, Canavarro MC. Does Hope Matter? Associations Among Self-Reported Hope, Anxiety, and Health-Related Quality of Life in Children and Adolescents with Cancer. J Clin Psychol Med Settings. 2018, 25(1):93-103.
47. Yu JJ, Chen YY, Liu CT, Oo Yang HJ. Correlation analysis of self-efficacy with anxiety, depression and quality of life in middle-aged and elderly patients with breast cancer undergoing chemotherapy. Clinical medical research and Practice, 2021,6(06):9-11.
48. Wu J, Wu SQ, Xiong WJ, Lv LH, Jiang J, Luo Jing, Rang WQ. Correlation between quality of life and hope, coping style in breast cancer patients. Practical Preventive Medicine. 2013, 20(4).
49. Zhang Y, Cui C, Wang Y, Wang L. Effects of stigma, hope and social support on quality of life among Chinese patients diagnosed with oral cancer: a cross-sectional study. Health Qual Life Outcomes. 2020, 18(1):112.
50. Zhou KN, Ning F, Wang W, Li XM. The mediator role of resilience between psychological predictors and health-related quality of life in breast cancer survivors: a cross-sectional study. Bmc Cancer 2022, 22(1).
51. Zhao YQ, Liu RY, Huo J. Correlation of self-feeling burden, self ⁃ efficacy and quality of life in patients with cervical cancer during radiotherapy. Chinese Nursing Research. 2020, 34(23):4159-4163.
52. Yeung NC, Lu Q. Affect as a mediator between self-efficacy and quality of life among Chinese cancer survivors in China. Eur J Cancer Care (Engl). 2014, 23(1):149-55.
53. Gong PF, Xia BR, Lou G, Wang WB, Zhang LY, Tian X, Liang XM, Zhou JW, Zhai H, Yang YJ. Mediating effect of hope level on correlation between perceived social support and quality of life among postoperative cervical cancer patients. Chin J Public Health. 2016, 32(7).
54. Chen YM, Yang YX, Yan SY, Ding XY, Yao H, Yao JP. Correlation between symptom distress, resilience and life quality in patients with breast cancer undergoing chemotherapy. Ningxia Med J. 2017, 39(9).
55. He YJ, Wang DM, Ma ZY. The association between mindfulness and quality of life in cancer patients: mediating role of resilience. Journal of MuDanJiang Medical University. 2019,40(01):127-129.
56. McAteer G, Gillanders D. Investigating the role of psychological flexibility, masculine self-esteem and stoicism as predictors of psychological distress and quality of life in men living with prostate cancer. Eur J Cancer Care (Engl). 2019, 28(4): e13097.
57. Sharour LA, Omari OA, Salameh AB, Yehia D. Health-related quality of life among patients with colorectal cancer. Journal of Research in Nursing. 2019.
58. Wong WS, Fielding R. Quality of life and pain in Chinese lung cancer patients: Is optimism a moderator or mediator? Qual Life Res. 2007, 16(1):53-63.
59. Omran S, Mcmillan S. Symptom Severity, Anxiety, Depression, Self- Efficacy and Quality of Life in Patients with Cancer. Asian Pac J Cancer Prev. 2018, 19(2):365-374.
60. Thieme M, Einenkel J, Zenger M, Hinz A. Optimism, pessimism and self-efficacy in female cancer patients. Jpn J Clin Oncol. 2017, 47(9):849-855.
61. Li MY, Yang YL, Liu L, Wang L. Effects of social support, hope and resilience on quality of life among Chinese bladder cancer patients: a cross-sectional study. Health Qual Life Outcomes. 2016,14:73.
62. Yuan J, Lin MY, Yang B, Shi LL, Zhao BY, Wang ZF, Chen YM. Mediating role of finical toxicity between self-efficacy and quality of life in patients with prostate cancer. Nurs J Chin PLA. 2022, 39(01):4-8.
63. Schofield PE, Stockler MR, Zannino D, Tebbutt NC, Price TJ, Simes RJ, Wong N, Pavlakis N, Ransom D, Moylan E, Underhill C, Wyld D, Burns I, Ward R, Wilcken N, Jefford M. Hope, optimism and survival in a randomised trial of chemotherapy for metastatic colorectal cancer. Support Care Cancer. 2016, 24(1):401-408.
64. Li Y, Zhou Z, Ni N, Li J, Luan Z, Peng X. Quality of Life and Hope of Women in China Receiving Chemotherapy for Breast Cancer. Clin Nurs Res. 2021, 14:10547738211046737.
65. Chen HL, Liu K, You QS. Self-efficacy, cancer-related fatigue, and quality of life in patients with resected lung cancer. Eur J Cancer Care (Engl). 2018, 27(6): e12934.
66. Vidthya S, Sherina MS, Rampal L, Fadhilah SI, Ummavathy P. Self-esteem among cancer patients receiving chemotherapy in selected. Med J Malaysia. 2019, 74(5):405-412.

**Figure A-C: Correlations between positive psychological resources and quality of life in cancer patients: Subgroup analysis.**


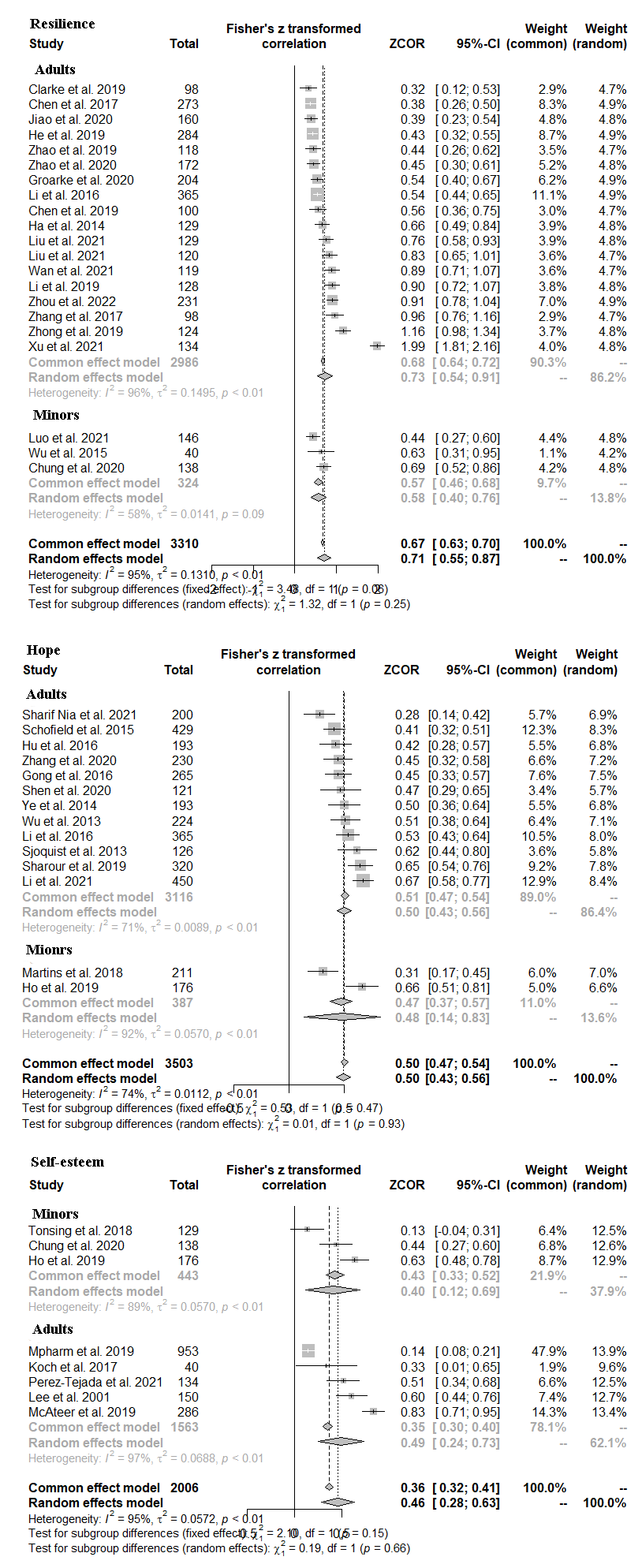
**A (Minors vs Adults)**

**B (Generic vs Specific instruments for assessing quality of life)**

**
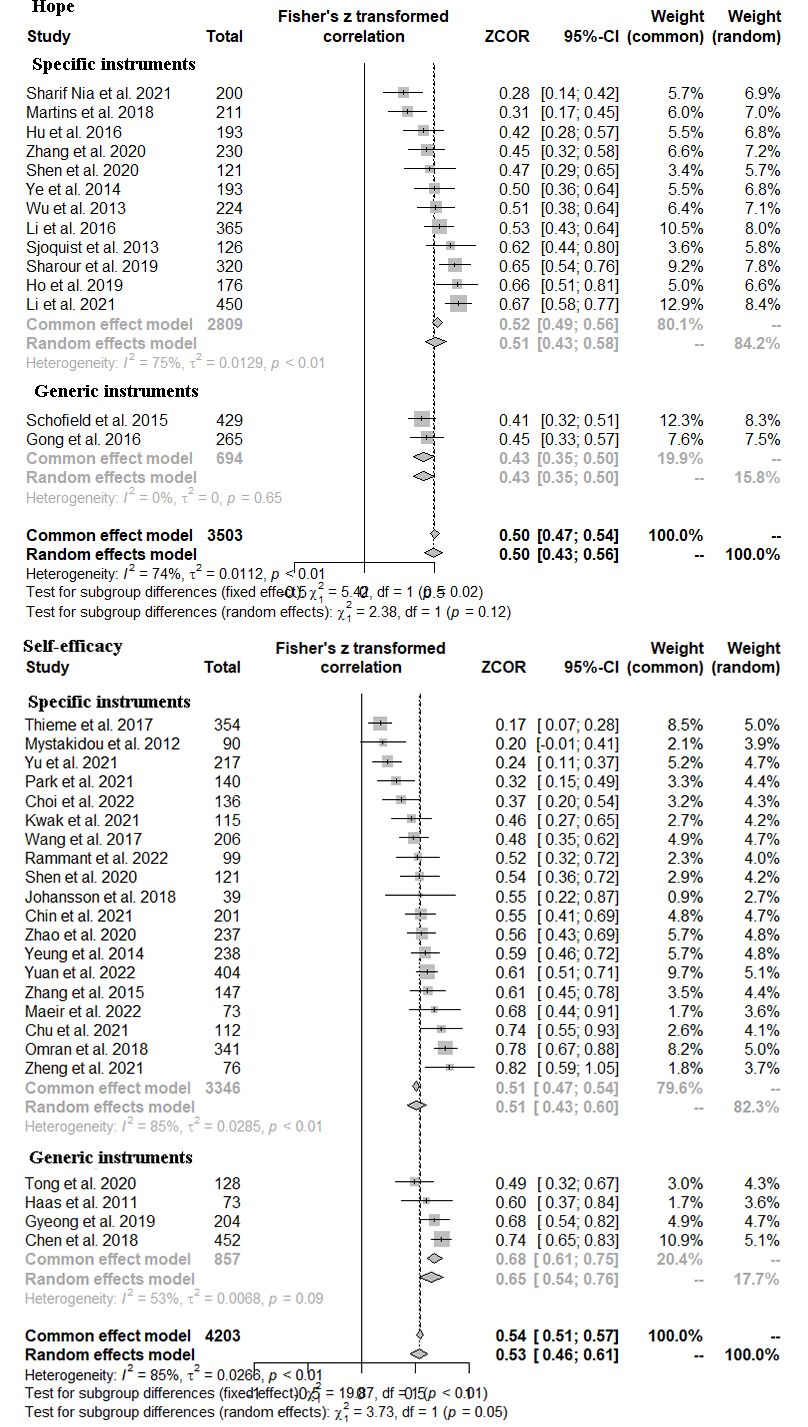
**

C (**Generic vs Specific instruments for assessing self-efficacy)**


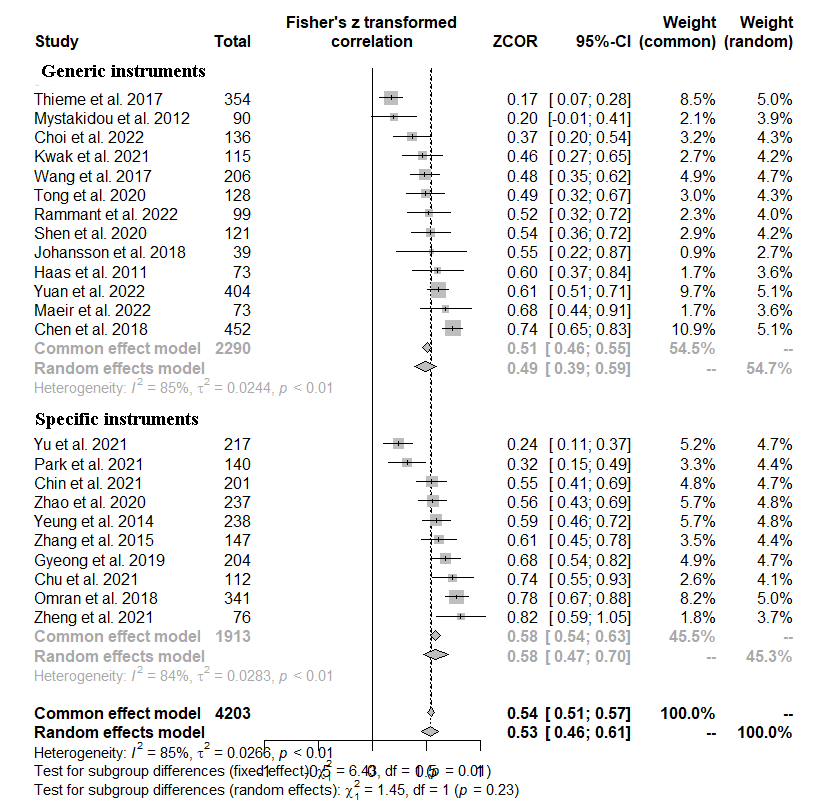

Supplement: Supplementary file 1 [file Data_Sheet_1.docx]
